# Improving on Half-Lightweight Male Judokas' High Performance by the Application of the Analytic Network Process

**DOI:** 10.3389/fpsyg.2021.621454

**Published:** 2021-04-07

**Authors:** Sugoi Uriarte Marcos, Raúl Rodríguez-Rodríguez, Juan-José Alfaro-Saiz, Eduardo Carballeira, Maier Uriarte Marcos

**Affiliations:** ^1^Doctorate School, Universitat Politècnica de València, Valencia, Spain; ^2^Department of Enterprises Management, Universitat Politècnica de València, Valencia, Spain; ^3^Performance and Health Group, Department of Physical Education and Sport Science, University of A Coruña, A Coruña, Spain; ^4^Faculty of Psychology and Education, University of Deusto, Bilbao, Spain

**Keywords:** performance improvements, judo, psychology skills, analytic network process, key performance indicators

## Abstract

Judo is a multifactorial sport where many variables or key performance indicators (KPIs) such as force-velocity profile, bioenergetic capacity, technical and tactical skills, and cognitive and emotional competence play a role and influence the final result. While there have been many academic studies of these variables, usually in isolation, none have examined KPIs holistically or analyzed their impact on strategic performance. The main objective of the present study, therefore, is to apply a novel and easily replicable methodology to identify and prioritize the main KPIs affecting performance in professional judo. Such a methodology was applied to the High-Performance Judo Centre of Valencia, using a multi-criteria decision aid technique: the analytic network process (ANP). The ANP is used to identify and quantify cause and effect relationships not only between KPIs but also between KPIs and performance objectives. Further, the ANP offers effective results when there is a lack of historical KPI data, because it is based on experts' opinions and judgments. A judo expert group (JEG) comprising elite judo coaches and half-lightweight (under 66 kg) male judokas applied the methodology to discriminate between the characteristics required when aiming to reach high-level strategic objectives (such as participating in the Olympic Games or winning a medal in a Grand Slam/Gran Prix). The JEG, which consisted of five elite judokas, national and international champions, and three Olympic coaches—including a former European champion and silver medalist in a world championship—provided high added value to the study. The main findings were that the KPIs that made the most difference were mostly psychological, specifically motivation, stress, and team cohesion. Of these, motivation was by far the most important KPI for success in our case study, so judokas should make sure that they analyze it properly. Motivation is usually intrinsic to the competitor and should be maintained at a high level, not only during tournaments but also during daily training and lifestyle activities. Physical and other specific forms of training, as well as lifestyle KPIs, are very important for the elite competitor but are not sufficient to reach high-level objectives. The most important of these KPIs were Kumi-Kata (grip work), dual career, focus and concentration level, scouting, nutrition, and basic technique. Power and strength were the most important physical KPIs. In general, these are essential for meeting strategic objectives, but they do not make the crucial difference. This suggests that professional psychological support should be provided in daily training and that international team composition and internships should be fostered.

## Introduction

There are currently many indicators to measure, control, and monitor sport performance. The recent incorporation and application of new data analysis techniques to professional sport (i.e., using big data or artificial intelligence) allow access to a more holistic analysis of the information extracted from real-world sports (Gu et al., 2019). This information is employed to enhance the decision-making process in the context of sports performance improvement, which should lead to goal achievement. Conversely, the coaching staff (i.e., coaches, sports psychologists, sports physicians, and so on) dispose of an overwhelming quantity of data that could help to establish what variables are key and which ones directly impact performance. Frequently, once the key performance indicators (KPIs) have been identified, problems arise when trying to identify which of these KPIs are directly linked to an athlete's ability to reach their strategic objectives. Such strategic objectives can be defined as the main goals that the athlete aims to achieve in the medium–long term (i.e., to obtain classification for the Olympic Games) and that therefore condition their efforts and planning. The strategic objectives should be few in number and realistically achievable to avoid frustration and wrongly defined preparation planning (Kaplan and Norton, 1992).

Competition in elite combat sports, such as boxing, tae-kwon-do, wrestling, and judo, requires complex technical skills with high strength and conditioning demands (Miarka et al., 2016). The athletes have to make correct decisions in a very short period of time, when multiple choices are available (Franchini et al., 2019). Furthermore, anxiety control and emotional intelligence are necessary not only on the day of competition but also in the preparation period, when athletes have to manage their weight and deal with a stressful training program (Merino Fernández et al., 2020). Previous studies have to some extent identified the variables that can be considered as KPIs for judo performance; these including weight management (Escobar-Molina et al., 2016; Thomson et al., 2017; Gallot et al., 2019), level of strength (Ache Dias et al., 2012; Franchini et al., 2019), technical and tactical skills (Weigelt et al., 2011; Bocioaca, 2014; Miarka et al., 2020), level and direction of motivation (Gillet et al., 2010; Boughattas et al., 2017; Oliveira et al., 2018), nutrition (Artioli et al., 2019), capacity for focus and attention (Yahija, 2010; Jacini et al., 2012; Mihailescu and Sava, 2013; Toh et al., 2018; Campos Faro et al., 2020), sleep habits (Chtourou et al., 2018; Knowles et al., 2018; Vlahoyiannis et al., in press), speed (Almansba et al., 2008), and level of conditioning capacity (aerobic and anaerobic; Detanico et al., 2012; Franchini et al., 2014, 2016; Hesari et al., 2014; Anthierens et al., 2016).

Recently, our research group (Uriarte Marcos et al., 2019) studied the KPIs for judo and sought to classify them into four clusters: general physical training, specific training tasks, lifestyle, and psychology skills. To the best of our knowledge, no scientific study has identified and prioritized the relative importance of KPIs in achieving strategic objectives in judo. The expected main benefits from prioritizing judo KPIs to achieve the athlete's strategic objectives include improvements in the design of training programs, identification of strengths and weakness of the athletes, saving and aligning efforts among technical staff working with judokas, and better organization at all levels. This should lead to higher performance. It is possible to find within the scientific literature studies that have dealt with linking judo KPIs with performance. Some authors have investigated the impact of weight loss on performance (Artioli et al., 2010; Fortes et al., 2017), while others have analyzed how the time of day influences explosive performance and psychological variables in elite judokas (Chtourou et al., 2018). The relationship between technical-tactical behaviors (Miarka et al., 2017) and physical capacities (Kons et al., 2018) has also been a focus of attention. Notwithstanding, the design of previous studies has limited researchers' capacity to explain the complexity of success in judo competition. They have invariably selected one or various KPIs and related them to outcomes in specific and unspecific test performances; this type of experimental design cannot eliminate the noise produced by strange variables when interpreting causality in performance outcomes. Employing an integrated approach, even at the risk of precision, could shed light on understanding the key skills needed for success in a complex multifaceted judo competition. Identifying the importance of particular KPIs might assist in the achievement of high-level strategic objectives. Experts' criteria have been used as a tool for detecting key performance indicators in sport (Sanchez et al., 2019). However, it has to be borne in mind that this technique involves a certain degree of subjectivity, so it is necessary to apply methods that manage this, for example multi-criteria decision aid (MCDA) techniques. In particular, the analytic network process (ANP; Saaty, 1996) captures the valuation of decision makers in a systematic and organized way and provides clear quantitative results. In addition, the original valuations can be easily modified if necessary, and at the same time, the results can be amended. Furthermore, the ANP has proven its effectiveness in identifying and quantifying the influence of heterogeneous KPIs on performance in similar research in other disciplines (Souza Farias et al., 2019; Andrade Arteaga et al., 2020).

Therefore, the main aims of the present study are as follows: (a) to identify and to prioritize the main KPIs that can help to build on the high performance of professional judokas; (b) to establish the link between judo KPIs and the achievement of strategic objectives; and (c) to make recommendations to improve performance based on the findings.

## Materials and Methods

### Participants

Five half-lightweight (under 66-kg category) male judokas and their coaches met the inclusion criteria for the judo expert group (JEG). They were recruited from the High-Performance Judo Centre of Valencia during a training camp. The inclusion criteria were that they should be elite judo athletes in the under 66-kg category with international presence and should have similar ambitious strategic objectives such as participation in the Olympic Games, international ranking improvement, or winning a Grand Prix/Slam. They were members of the national teams of Spain, Italy, USA, France, the Dominican Republic, and Puerto Rico and represented their countries as athletes and national coaches in international championships. Their personal data and assessments were coded during the research process to ensure that all information was anonymous and protected.

### Procedure

**Figure 1:**
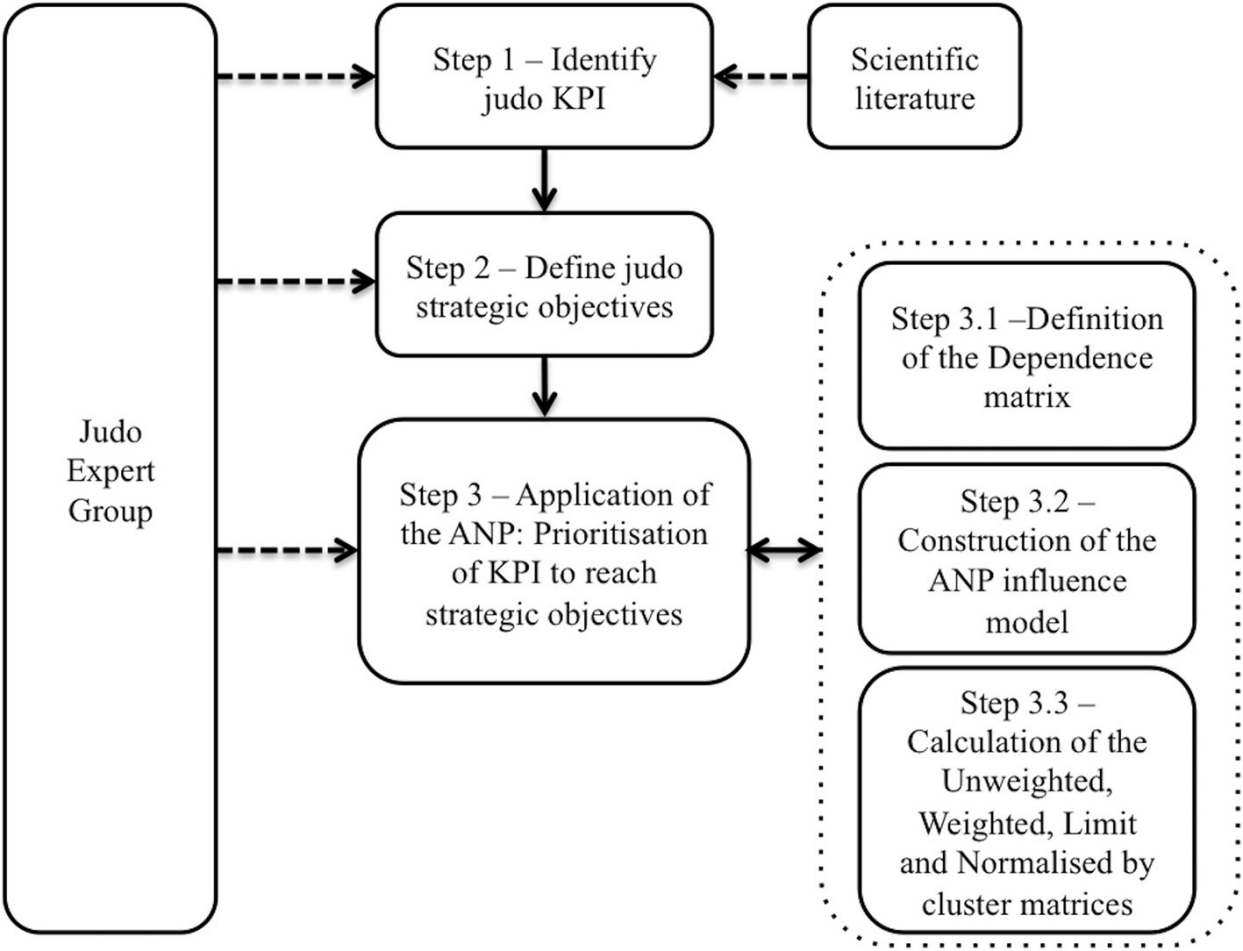
Methodology.

The methodology used to prioritize the KPIs for professional judokas to reach strategic objectives is presented in Figure 1.

#### Step 1: Identify Main Judo KPI

The objective of this step was to identify the main judo KPI using the process employed in the scientific literature and the experience of the JEG. The JEG first used the classification carried out by Uriarte Marcos et al. (2019), as this work not only identified the most important KPIs for elite judo athletes but also classified them into four clusters (physical training, specific training, psychology, and lifestyle). They then decided by consensus which KPI from the literature should be kept for the purposes of the research and which others to add. The KPIs were selected according to their capacity to impact the judo athlete's high performance.

#### Step 2: Define Judo Strategic Objectives

In this step, the JEG defined the strategic objectives for the judokas (with the researchers' assistance). The members were asked to write down freely the five medium-term (i.e., the next 2–3 years) strategic objectives they regarded as the most important. These were then analyzed, and it was agreed to keep four of them based on their frequency of appearance in the survey and on their alignment.

#### Step 3: Application of the ANP: Prioritization of KPIs According to the Strategic Objectives

In this step, the KPIs were prioritized according to their importance in achieving the strategic objectives by applying the analytic network process (ANP; Saaty, 1996). The main steps of the ANP, and how the JEG developed them, are presented next.

##### Step 3.1: Definition of the Dependence Matrix

This involved the JEG identifying any potential relationships between each pair of variables and forming a one–zero matrix called the dependence matrix, which indicates the presence of a meaningful relationship between the two (Saaty, 1996). Additionally, the variables were grouped in different clusters according to their common characteristics; e.g., the physical training KPI constituted the “physical training cluster.”

##### Step 3.2: Construction of the ANP Influence Model

The results of the dependence matrix were used to build the ANP influence model, which is a graphical representation of the matrix, where the relationships established between both clusters and elements are introduced.

##### Step 3.3: Construction of the Unweighted, Weighted, Limit, and Normalized-by-Cluster Matrices

Different matrices were worked out to determine the influence of the different elements of the ANP model (i.e., the KPIs and strategic objectives). The JEG began by completing a pair-wise questionnaire for all the existing relationships defined in the dependence matrix and modeled in the ANP influence model. They then compared two elements according to their influence on a third and quantified the influence using Saaty's Fundamental Scale (see Table 1).

**Table 1:** Saaty's Fundamental Scale (Saaty, 1996).

**Intensity of importance**	**Definition**
1	Equal importance/preference
2	Weak
3	Moderate importance/preference
4	Moderate plus
5	Strong importance/preference
6	Strong plus
7	Very strong or demonstrated importance/preference
8	Very, very strong
9	Extreme importance/preference

For example, the question “Which element has a greater influence on the ‘Olympic Games classification’ strategic objective: ‘Technical and tactical skills’ or ‘Relative strength’?” had to be scored as a 5 if the judo expert valued that the KPI “Technical and tactical skills” was substantially more important that the KPI “Relative strength” in achieving the strategic objective of “Olympic Games classification.”

Once the pair-wise comparisons were made, the first matrix computed was the unweighted matrix, followed by the weighted matrix. The latter made it possible to extract directly the cause and effect relationships between each pair of elements of the network and to identify the KPIs that strongly affected the strategic objectives. The matrix was then raised to as many powers as were necessary to obtain the limit matrix, in which all of the columns would amount to 100%. The limit matrix offered the global relative weight of each variable and, in the present study, provided the basis for the prioritization not only of the KPIs but also the strategic objectives. Finally, the normalized matrix was computed; this showed the relative importance of each variable within its cluster.

Derived from these matrices, it is possible to conduct different analyses. Then, in this research, three analyses are carried out:

1. Cause–effect analysis. This analysis is made from the results of the unweighted matrix and it identifies the main direct cause–effect relationships between each pair of variables. Then, it aims to determine the most important cause KPIs that are influencing not only to other KPIs but also to the strategic objectives. In general, these KPIs that are producing a high intensity and direct effect on the strategic objectives should be fostered, as the main aim of the judokas is to reach such objectives. Additionally, this analysis also studies the cause–effect relationships between the strategic objectives, being then able to classify and align such objectives. Finally, it is also possible to study the indirect cause–effect relationships that would identify the KPIs that are strongly influencing to other KPIs, which at the same time strongly influence to the strategic objectives.
2. ABC analysis. This analysis is based on the results of the limit matrix and categorizes the study variables as follows:
  - Class A includes the variables that account for ~65–70% of the total value of the limit matrix. These are the most important variables of the network from a global point of view;
  - Class B includes the variables that account for ~20% of the total value of the limit matrix. The importance of the B class variables is medium;
  - Class C includes the variables that account for ~10–15% of the total value of the limit matrix. The importance of the C class variables is low.
3. Cluster analysis. This analysis is based on the normalized-by-cluster matrix and identifies, for each of the defined clusters, the most important variables, ranking them and offering a cluster-based vision for decision-makers.

All comparisons and posterior matrices outcomes were processed using the software Superdecisions V2.10 (www.superdecisions.com, Pittsburg, USA).

## Results

### Step 1: Identifying KPIs for Judo

The JEG retained all 15 KPIs classified into the four clusters defined previously (Uriarte Marcos et al., 2019) and modified some of them. They also added other KPIs that they felt had an important impact on the high performance of male half-lightweight judokas in reaching their high-level strategic objectives. There were a total of 26 KPIs in all. Table 2 summarizes both the KPIs and clusters used in the study and the original ones defined by Uriarte Marcos et al. (2019).

**Table 2:** Selection of the judo KPI and clusters.

**Cluster**	**KPI from Uriarte Marcos et al. (2019)**	**KPI for research**
Physical training	Coordination	
	Strength	Maximum strength Relative strength
	Speed	
	Heart rate	Cardiac frequency—basal Cardiac recovery
	Aerobic and anaerobic fitness	Aerobic resistance Anaerobic resistance
	Flexibility	
	Power	
Specific training	Technical and tactical preparation	Basic tactic Basic technique Specific tactic Specific technique
	Age	
	Weight	
	Kumi-Kata	
	Scouting	
Psychology	Focus and concentration level	
	Stress	
	Motivation	
	Activation level	Activation level
	Team cohesion	
Lifestyle	Nutrition	
	Dual career	
	Sleep	8/8/8

### Step 2: Define Judo Strategic Objectives

The JEG drew up a list of nine strategic objectives, from which the following four were retained for the research:

- To qualify for the Olympic Games;
- To win a medal in either a Grand Prix or a Grand Slam;
- To make a significant improvement in international ranking;
- To become (or remain) national champion.

### Step 3: Application of the ANP: Prioritization of KPIs to Reach the Strategic Objectives

#### Step 3.1: Definition of the Dependence Matrix

Table 3 shows the definition of the dependence matrix.

Table 3Dependence matrix.

**Table:** 

		**AR**	**ANR**	**CF**	**CR**	**MaxS**	**RelS**	**Sp**	**Pw**	**Coor**	**Flex**
**DEPENDENCE MATRIX (1/3)**											
Physical training	Aerobic resistance (AR)	0	1	1	1	0	0	0	0	1	0
	Anaerobic resistance (ANR)	1	0	1	1	0	0	1	1	1	0
	Cardiac frequency—basal (CF)	1	1	0	1	0	0	0	0	0	0
	Cardiac recovery (CR)	1	1	1	0	0	0	1	1	0	0
	Maximum strength (MaxS)	0	0	0	0	0	1	0	1	0	0
	Relative strength (RelS)	0	0	0	0	1	0	0	1	0	0
	Speed (Sp)	1	1	0	0	0	0	0	1	0	0
	Power (Pw)	1	1	0	0	1	1	1	0	0	0
	Coordination (Coor)	0	0	0	0	1	1	1	1	0	0
	Flexibility (Flex)	0	0	0	0	0	0	0	0	1	0
Specific training	Basic technique (BT)	0	0	0	0	1	1	0	0	1	1
	Specific technique (ST)	0	0	0	0	1	1	0	0	1	1
	Basic tactic (BTac)	0	0	0	0	0	0	0	0	0	0
	Specific tactic (Stac)	0	0	0	0	0	0	0	0	0	0
	Kumi-Kata (KK)	0	0	0	0	1	1	1	1	1	0
	Scouting (SC)	0	0	0	0	0	0	0	0	0	0
	Weight (Wei)	1	1	1	1	1	1	1	1	1	0
	Age	1	1	1	1	1	1	1	1	1	1
Lifestyle	Nutrition (Nut)	1	1	1	1	1	1	1	1	1	0
	Dual career (Dual)	0	0	0	0	0	0	0	0	0	0
	8/8/8	1	1	1	1	1	1	1	1	0	0
Psychology	Motivation (Mot)	1	1	1	1	1	1	1	1	1	1
	Stress (Stress)	1	1	1	1	1	1	1	1	1	0
	Activation level (Act)	0	0	1	0	1	1	1	1	1	0
	Focus and concentration level (Foc)	0	0	0	0	1	1	1	1	1	0
	Team cohesion (Cohe)	0	0	0	0	0	0	0	0	0	0
Objectives	National champion (N. Champ)	0	0	0	0	0	0	0	0	0	0
	International ranking improvement (IRI)	0	0	0	0	0	0	0	0	0	0
	GP/GS medal	0	0	0	0	0	0	0	0	0	0
	OG classification	0	0	0	0	0	0	0	0	0	0

**Table:** 

		**Specific training**								**Lifestyle**		
		**BT**	**ST**	**Btac**	**Stac**	**KK**	**SC**	**Wei**	**Age**	**Nut**	**Dual**	**8/8/8**
**DEPENDENCE MATRIX (2/3)**												
Physical training	Aerobic resistance (AR)	0	1	0	1	1	0	1	0	0	0	0
	Anaerobic resistance (ANR)	0	1	0	1	1	0	1	0	0	0	0
	Cardiac frequency—basal (CF)	0	0	0	0	0	0	0	0	0	0	0
	Cardiac recovery (CR)	0	1	0	1	0	0	0	0	0	0	0
	Maximum strength (MaxS)	1	1	0	1	1	0	1	0	0	0	0
	Relative strength (RelS)	1	1	0	1	1	0	0	0	0	0	0
	Speed (Sp)	1	1	0	1	1	0	0	0	0	0	0
	Power (Pw)	1	1	0	1	1	0	0	0	0	0	0
	Coordination (Coor)	1	1	1	1	1	0	0	0	0	0	0
	Flexibility (Flex)	1	1	0	0	0	0	0	0	0	0	0
Specific training	Basic technique (BT)	0	1	1	1	1	1	0	0	0	0	0
	Specific technique (ST)	1	0	1	1	1	1	0	0	0	0	0
	Basic tactic (BTac)	1	1	0	1	1	1	0	0	0	0	0
	Specific tactic (Stac)	1	1	1	0	1	1	0	0	0	0	0
	Kumi-Kata (KK)	1	1	1	1	0	1	0	0	0	0	0
	Scouting (SC)	1	1	1	1	1	0	0	0	0	0	0
	Weight (Wei)	1	1	1	1	1	1	0	0	1	1	1
	Age	1	1	1	1	1	1	1	0	1	1	1
Lifestyle	Nutrition (Nut)	1	1	1	1	1	0	1	0	0	1	1
	Dual career (Dual)	0	0	0	0	0	1	1	0	1	0	1
	8/8/8	1	1	1	1	1	1	1	0	1	1	0
Psychology	Motivation (Mot)	1	1	1	1	1	1	1	0	1	1	1
	Stress (Stress)	1	1	1	1	1	1	1	0	1	1	1
	Activation level (Act)	1	1	1	1	1	1	1	0	1	1	1
	Focus and concentration level (Foc)	1	1	1	1	1	1	1	0	1	1	1
	Team cohesion (Cohe)	1	1	1	1	1	1	1	0	1	1	1
Objectives	National champion (N. Champ)	0	0	0	0	0	0	0	0	0	0	0
	International ranking improvement (IRI)	0	0	0	0	0	0	0	0	0	0	0
	GP/GS medal	0	0	0	0	0	0	0	0	0	0	0
	OG classification	0	0	0	0	0	0	0	0	0	0	0

**Table:** 

		**Psychology**					**Objectives**			
		**Mot**	**Stress**	**Act**	**Foc**	**Cohe**	**N champ**	**IRI**	**Medal**	**OG**
**DEPENDENCE MATRIX (3/3)**										
Physical training	Aerobic resistance (AR)	1	1	0	0	0	1	1	1	1
	Anaerobic resistance (ANR)	1	1	0	0	0	1	1	1	1
	Cardiac frequency—basal (CF)	0	0	0	0	0	1	1	1	1
	Cardiac recovery (CR)	1	1	0	1	0	1	1	1	1
	Maximum strength (MaxS)	1	1	0	0	0	1	1	1	1
	Relative strength (RelS)	1	1	0	0	0	1	1	1	1
	Speed (Sp)	1	1	0	0	0	1	1	1	1
	Power (Pw)	1	1	0	0	0	1	1	1	1
	Coordination (Coor)	1	1	0	0	0	1	1	1	1
	Flexibility (Flex)	1	1	0	0	0	1	1	1	1
Specific training	Basic technique (BT)	1	1	0	0	0	1	1	1	1
	Specific technique (ST)	1	1	0	0	0	1	1	1	1
	Basic tactic (BTac)	1	1	0	1	0	1	1	1	1
	Specific tactic (Stac)	1	1	0	1	0	1	1	1	1
	Kumi-Kata (KK)	1	1	0	1	0	1	1	1	1
	Scouting (SC)	1	1	0	1	0	1	1	1	1
	Weight (Wei)	1	1	1	1	1	1	1	1	1
	Age	1	1	1	1	1	1	1	1	1
Lifestyle	Nutrition (Nut)	1	1	1	1	0	1	1	1	1
	Dual career (Dual)	1	1	0	0	1	1	1	1	1
	8/8/8	1	1	0	0	1	1	1	1	1
Psychology	Motivation (Mot)	0	1	1	1	1	1	1	1	1
	Stress (Stress)	1	0	1	1	1	1	1	1	1
	Activation level (Act)	1	1	0	1	1	1	1	1	1
	Focus and concentration level (Foc)	1	1	1	0	1	1	1	1	1
	Team cohesion (Cohe)	1	1	1	1	0	1	1	1	1
Objectives	National champion (N. Champ)	0	0	0	0	0	0	0	0	1
	International ranking improvement (IRI)	0	0	0	0	0	0	0	0	0
	GP/GS medal	0	0	0	0	0	0	1	0	1
	OG classification	0	0	0	0	0	1	0	1	0

*The JEG decided whether the relationship between each pair of variables was either significant (one) or not (zero). This Table has been divided, due to space limitations, into three parts*.

#### Step 3.2: Construction of the ANP Influence Model

**Figure 2:**
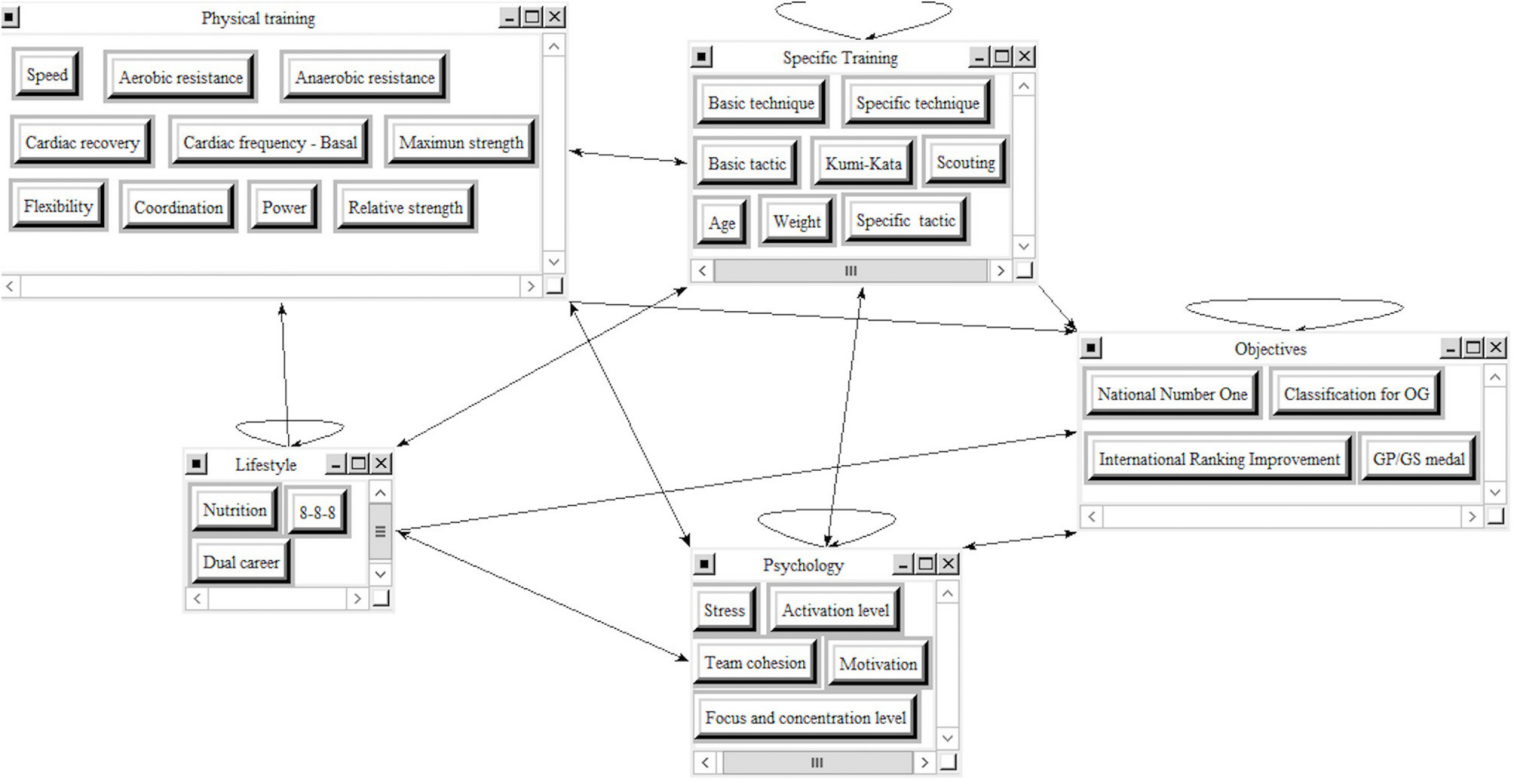
ANP influence model in Superdecisions TM.

We designed the ANP influence model (Figure 2), which contains all the variables, clusters, and their relationships as defined in the dependence matrix.

#### Step 3.3: Construction of the Unweighted, Weighted, Limit, and Normalized-by-Cluster Matrices

The first activity was to state the influence between each pair of variables and to reach a third using Saaty's Fundamental Scale. The unweighted matrix and the weighted matrix (Table 4) were thus obtained.

Table 4Weighted matrix.

**Table:** 

		**Lifestyle**			**Objectives**			
		**8/8/8**	**Dual**	**Nut**	**OG**	**Medal**	**IRI**	**N champ**
**WEIGHTED MATRIX (1/4)**								
Lifestyle	8/8/08	0.00	0.24	0.24	0.00	0.00	0.00	0.00
	Dual career (Dual)	0.22	0.00	0.08	0.00	0.00	0.00	0.00
	Nutrition (Nut)	0.11	0.12	0.00	0.00	0.00	0.00	0.00
Objectives	OG classification	0.06	0.07	0.07	0.00	0.22	0.00	0.22
	GP/GS medal	0.04	0.04	0.03	0.29	0.00	0.33	0.00
	International ranking improvement (IRI)	0.03	0.03	0.02	0.00	0.11	0.00	0.11
	National champion (N. Champ)	0.02	0.02	0.02	0.04	0.00	0.00	0.00
Physical training	Aerobic resistance (AR)	0.02	0.00	0.02	0.00	0.00	0.00	0.00
	Anaerobic resistance (ANR)	0.02	0.00	0.02	0.00	0.00	0.00	0.00
	Cardiac frequency—basal (CF)	0.01	0.00	0.01	0.00	0.00	0.00	0.00
	Cardiac recovery (CR)	0.02	0.00	0.01	0.00	0.00	0.00	0.00
	Coordination (Coor)	0.00	0.00	0.01	0.00	0.00	0.00	0.00
	Flexibility (Flex)	0.00	0.00	0.00	0.00	0.00	0.00	0.00
	Maximum strength (MaxS)	0.01	0.00	0.01	0.00	0.00	0.00	0.00
	Power (Pw)	0.01	0.00	0.01	0.00	0.00	0.00	0.00
	Relative strength (RelS)	0.01	0.00	0.01	0.00	0.00	0.00	0.00
	Speed (Sp)	0.01	0.00	0.01	0.00	0.00	0.00	0.00
Psychology	Activation level (Act)	0.00	0.00	0.01	0.00	0.00	0.00	0.00
	Focus and concentration level (Foc)	0.12	0.00	0.02	0.00	0.00	0.00	0.00
	Motivation (Mot)	0.07	0.15	0.15	0.36	0.36	0.36	0.00
	Stress (Stress)	0.04	0.08	0.04	0.20	0.20	0.20	0.67
	Team cohesion (Cohe)	0.02	0.04	0.03	0.11	0.11	0.11	0.00
Specific training	Age	0.00	0.00	0.01	0.00	0.00	0.00	0.00
	Basic technique (BT)	0.03	0.00	0.01	0.00	0.00	0.00	0.00
	Basic tactic (BTac)	0.04	0.00	0.01	0.00	0.00	0.00	0.00
	Kumi-Kata (KK)	0.05	0.00	0.01	0.00	0.00	0.00	0.00
	Scouting (SC)	0.03	0.07	0.00	0.00	0.00	0.00	0.00
	Specific technique (ST)	0.02	0.00	0.01	0.00	0.00	0.00	0.00
	Specific tactic (Stac)	0.02	0.00	0.01	0.00	0.00	0.00	0.00
	Weight (Wei)	0.00	0.14	0.11	0.00	0.00	0.00	0.00

**Table:** 

		**Physical training**									
		**AR**	**ANR**	**CF**	**CR**	**Coor**	**Flex**	**MaxS**	**Pw**	**RelS**	**Sp**
**WEIGHTED MATRIX (2/4)**											
Lifestyle	8/8/8	0.00	0.00	0.00	0.00	0.00	0.00	0.00	0.00	0.00	0.00
	Dual career (Dual)	0.00	0.00	0.00	0.00	0.00	0.00	0.00	0.00	0.00	0.00
	Nutrition (Nut)	0.00	0.00	0.00	0.00	0.00	0.00	0.00	0.00	0.00	0.00
Objectives	OG classification	0.12	0.14	0.16	0.11	0.11	0.11	0.11	0.14	0.11	0.11
	GP/GS medal	0.07	0.06	0.11	0.08	0.08	0.08	0.08	0.09	0.08	0.08
	International ranking improvement (IRI)	0.05	0.05	0.08	0.05	0.05	0.05	0.05	0.05	0.05	0.05
	National champion (N. Champ)	0.03	0.03	0.06	0.04	0.04	0.04	0.04	0.00	0.04	0.04
Physical training	Aerobic resistance (AR)	0.00	0.06	0.38	0.12	0.00	0.00	0.00	0.02	0.00	0.03
	Anaerobic resistance (ANR)	0.06	0.00	0.07	0.05	0.00	0.00	0.00	0.03	0.00	0.27
	Cardiac frequency—basal (CF)	0.19	0.04	0.00	0.16	0.00	0.00	0.00	0.00	0.00	0.00
	Cardiac recovery (CR)	0.11	0.03	0.13	0.00	0.00	0.00	0.00	0.00	0.00	0.00
	Coordination (Coor)	0.02	0.05	0.00	0.00	0.00	0.39	0.00	0.00	0.00	0.00
	Flexibility (Flex)	0.00	0.00	0.00	0.00	0.00	0.00	0.00	0.00	0.00	0.00
	Maximum strength (MaxS)	0.00	0.00	0.00	0.00	0.04	0.00	0.00	0.17	0.26	0.00
	Power (Pw)	0.00	0.11	0.00	0.03	0.10	0.00	0.26	0.00	0.13	0.09
	Relative strength (RelS)	0.00	0.00	0.00	0.00	0.06	0.00	0.13	0.13	0.00	0.00
	Speed (Sp)	0.00	0.10	0.00	0.03	0.18	0.00	0.00	0.04	0.00	0.00
Psychology	Activation level (Act)	0.00	0.00	0.00	0.00	0.00	0.00	0.00	0.00	0.00	0.00
	Focus and concentration level (Foc)	0.00	0.00	0.00	0.06	0.00	0.00	0.00	0.00	0.00	0.00
	Motivation (Mot)	0.17	0.15	0.00	0.03	0.17	0.17	0.16	0.16	0.16	0.15
	Stress (Stress)	0.02	0.05	0.00	0.11	0.02	0.02	0.04	0.03	0.03	0.05
	Team cohesion (Cohe)	0.00	0.00	0.00	0.00	0.00	0.00	0.00	0.00	0.00	0.00
Specific training	Age	0.00	0.00	0.00	0.00	0.00	0.00	0.00	0.00	0.00	0.00
	Basic technique (BT)	0.00	0.00	0.00	0.00	0.01	0.00	0.00	0.03	0.00	0.02
	Basic tactic (BTac)	0.00	0.00	0.00	0.00	0.05	0.09	0.02	0.05	0.03	0.04
	Kumi-Kata (KK)	0.04	0.05	0.00	0.00	0.02	0.00	0.06	0.00	0.07	0.05
	Scouting (SC)	0.00	0.00	0.00	0.00	0.00	0.00	0.00	0.00	0.00	0.00
	Specific technique (ST)	0.01	0.02	0.00	0.05	0.02	0.00	0.01	0.02	0.02	0.01
	Specific tactic (Stac)	0.01	0.02	0.00	0.09	0.04	0.05	0.01	0.05	0.02	0.02
	Weight (Wei)	0.07	0.04	0.00	0.00	0.00	0.00	0.03	0.00	0.00	0.00

**Table:** 

		**Psychology**				
		**Act**	**Foc**	**Mot**	**Stress**	**Cohe**
**WEIGHTED MATRIX (3/4)**						
Lifestyle	8/8/8	0.05	0.10	0.05	0.05	0.03
	Dual career (Dual)	0.03	0.05	0.10	0.10	0.11
	Nutrition (Nut)	0.10	0.03	0.03	0.03	0.06
Objectives	OG classification	0.09	0.09	0.09	0.18	0.10
	GP/GS medal	0.07	0.07	0.07	0.02	0.07
	International ranking improvement (IRI)	0.05	0.05	0.05	0.01	0.05
	National champion (N. Champ)	0.03	0.03	0.03	0.03	0.04
Physical training	Aerobic resistance (AR)	0.00	0.00	0.01	0.01	0.00
	Anaerobic resistance (ANR)	0.00	0.00	0.01	0.01	0.00
	Cardiac frequency—basal (CF)	0.02	0.03	0.00	0.00	0.00
	Cardiac recovery (CR)	0.00	0.00	0.00	0.01	0.00
	Coordination (Coor)	0.02	0.01	0.01	0.01	0.00
	Flexibility (Flex)	0.00	0.00	0.00	0.00	0.00
	Maximum strength (MaxS)	0.02	0.01	0.02	0.02	0.00
	Power (Pw)	0.01	0.02	0.02	0.02	0.00
	Relative strength (RelS)	0.01	0.02	0.02	0.02	0.00
	Speed (Sp)	0.02	0.01	0.01	0.01	0.00
Psychology	Activation level (Act)	0.00	0.07	0.04	0.04	0.05
	Focus and concentration level (Foc)	0.17	0.00	0.06	0.10	0.07
	Motivation (Mot)	0.09	0.14	0.21	0.15	0.16
	Stress (Stress)	0.05	0.09	0.00	0.00	0.10
	Team cohesion (Cohe)	0.03	0.04	0.03	0.06	0.00
Specific training	Age	0.00	0.00	0.00	0.00	0.00
	Basic technique (BT)	0.01	0.01	0.02	0.01	0.02
	Basic tactic (BTac)	0.03	0.02	0.03	0.02	0.03
	Kumi-Kata (KK)	0.02	0.03	0.03	0.03	0.04
	Scouting (SC)	0.04	0.02	0.01	0.02	0.01
	Specific technique (ST)	0.02	0.01	0.01	0.01	0.01
	Specific tactic (Stac)	0.01	0.02	0.02	0.01	0.02
	Weight (Wei)	0.01	0.01	0.01	0.04	0.01

**Table:** 

		**Specific training**							
		**Age**	**BT**	**Btac**	**KK**	**SC**	**ST**	**Stac**	**Wei**
**WEIGHTED MATRIX (4/4)**									
Lifestyle	8/8/8	0.02	0.00	0.00	0.00	0.00	0.00	0.00	0.01
	Dual career (Dual)	0.03	0.00	0.00	0.00	0.00	0.00	0.00	0.01
	Nutrition (Nut)	0.05	0.00	0.00	0.00	0.00	0.00	0.00	0.08
Objectives	OG classification	0.06	0.12	0.10	0.10	0.12	0.12	0.10	0.09
	GP/GS medal	0.06	0.08	0.07	0.07	0.08	0.08	0.07	0.06
	International ranking improvement (IRI)	0.06	0.06	0.05	0.05	0.06	0.06	0.05	0.05
	National champion (N. Champ)	0.06	0.04	0.04	0.04	0.04	0.04	0.04	0.03
Physical training	Aerobic resistance (AR)	0.01	0.00	0.00	0.00	0.00	0.00	0.00	0.04
	Anaerobic resistance (ANR)	0.01	0.00	0.00	0.00	0.00	0.00	0.00	0.03
	Cardiac frequency—basal (CF)	0.01	0.00	0.00	0.00	0.00	0.00	0.00	0.01
	Cardiac recovery (CR)	0.01	0.00	0.00	0.00	0.00	0.00	0.00	0.02
	Coordination (Coor)	0.01	0.00	0.07	0.01	0.00	0.00	0.07	0.01
	Flexibility (Flex)	0.01	0.00	0.04	0.00	0.00	0.00	0.04	0.00
	Maximum strength (MaxS)	0.01	0.00	0.02	0.03	0.00	0.00	0.01	0.01
	Power (Pw)	0.01	0.00	0.00	0.02	0.00	0.00	0.00	0.01
	Relative strength (RelS)	0.01	0.00	0.03	0.05	0.00	0.00	0.02	0.01
	Speed (Sp)	0.01	0.00	0.00	0.04	0.00	0.00	0.00	0.01
Psychology	Activation level (Act)	0.03	0.00	0.00	0.00	0.00	0.00	0.00	0.01
	Focus and concentration level (Foc)	0.03	0.05	0.00	0.04	0.04	0.05	0.00	0.03
	Motivation (Mot)	0.03	0.14	0.17	0.13	0.17	0.15	0.15	0.07
	Stress (Stress)	0.03	0.03	0.02	0.02	0.00	0.02	0.04	0.02
	Team cohesion (Cohe)	0.03	0.00	0.00	0.00	0.00	0.00	0.00	0.04
Specific training	Age	0.00	0.00	0.00	0.00	0.00	0.00	0.00	0.00
	Basic technique (BT)	0.05	0.00	0.05	0.07	0.08	0.08	0.03	0.03
	Basic tactic (BTac)	0.05	0.06	0.00	0.05	0.04	0.03	0.12	0.07
	Kumi-Kata (KK)	0.05	0.17	0.02	0.00	0.18	0.19	0.05	0.17
	Scouting (SC)	0.05	0.12	0.18	0.15	0.00	0.13	0.17	0.02
	Specific technique (ST)	0.05	0.08	0.04	0.09	0.11	0.00	0.02	0.04
	Specific tactic (Stac)	0.05	0.04	0.11	0.04	0.06	0.05	0.00	0.05
	Weight (Wei)	0.05	0.00	0.00	0.00	0.00	0.00	0.00	0.00

*This Table has been divided, due to space limitations, into four parts*.

### Cause–Effect Analysis

**Figure 3:**
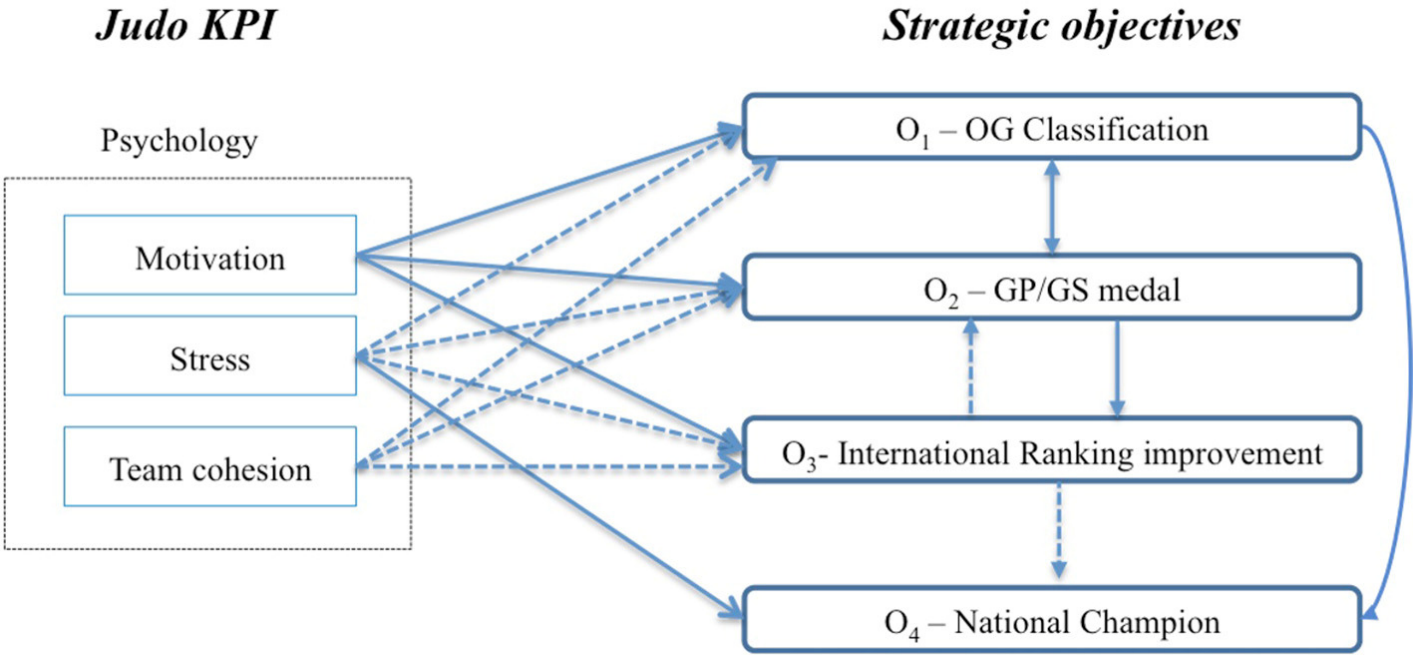
Impact of KPI on the strategic objectives.

Using the weighted matrix, it was possible to identify which KPI directly influenced the strategic objectives the most, as well as the relationships between the latter. The variables in the rows represent the cause and the variables in the columns the effects (Table 4). This leads to the construction of Figure 3, which shows that three KPIs—motivation, stress, and team cohesion—directly and meaningfully affected the strategic objectives (O_1_, O_2_, O_3_, and O_4_). Some of the effects were of greater intensity (continuous arrows) than others (discontinuous arrows). They are described as follows:

- Motivation strongly influences O_1_ (OG qualification), O_2_ (GP/GS medal), and O_3_ (international ranking improvement);
- Stress strongly influences O_4_ (national champion) and moderately influences O_1_, O_2_, and O_3_;
- Team cohesion has a moderate influence on O_1_, O_2_, and O_3_.

When considering the strategic objectives, it is also possible to find the following:

- There is a dual and strong relationship between the priority objectives; reaching O_1_ will help one to reach O_2_ and vice versa;
- There is a strong relationship between O_2_ and O_3_ (i.e., winning a medal will lead to an improvement in international ranking) and a moderate relationship between O_3_ and O_2_ (i.e., an improvement in international ranking will not necessarily lead to winning a medal);
- There is a moderate relationship between O_2_ and O_1_ (i.e., an improvement in international ranking is aligned with becoming a national champion);
- There is a strong relationship between O_1_ and O_4_ (i.e., participating in the Olympic Games helps the competitor to become a national champion).

### ABC Analysis

The limit matrix is shown in Table 5, along with an ABC analysis that categorizes the study variables as specified previously in the paper.

**Table 5:** Limit matrix and ABC analysis.

**Cluster**	**Variable**	**Limit**	**Cumulative percentage**	**Class**
Psychology	Motivation	0.20158	20%	A
Objectives	Classification for OG	0.10362	31%	A
Objectives	GP/GS medal	0.08958	39%	A
Psychology	Stress	0.08664	48%	A
Lifestyle	Dual career	0.04588	53%	A
Objectives	International ranking improvement	0.04434	57%	A
Psychology	Team cohesion	0.04235	61%	A
Lifestyle	8/8/8	0.03878	65%	B
Psychology	Focus and concentration level	0.03721	69%	B
Specific training	Kumi-Kata	0.03262	72%	B
Objectives	National number one	0.02762	75%	B
Specific training	Scouting	0.02622	78%	B
Lifestyle	Nutrition	0.02487	80%	B
Specific training	Basic technique	0.02043	82%	B
Psychology	Activation level	0.01704	84%	C
Specific training	Specific technique	0.01699	86%	C
Specific training	Basic tactic	0.01672	87%	C
Physical training	Maximum strength	0.01653	89%	C
Specific training	Weight	0.01647	91%	C
Specific training	Specific tactic	0.01645	92%	C
Physical training	Power	0.01606	94%	C
Physical training	Relative strength	0.01477	95%	C
Physical training	Aerobic resistance	0.00942	96%	C
Physical training	Anaerobic resistance	0.00872	97%	C
Physical training	Speed	0.00809	98%	C
Physical training	Coordination	0.00708	99%	C
Physical training	Cardiac frequency—basal	0.0065	99%	C
Physical training	Cardiac recovery	0.00552	100%	C
Physical training	Flexibility	0.00154	100%	C
Specific training	Age	0.00036	100%	C

The A class KPIs were motivation, stress management, team cohesion, and dual career. The last three belonged to the psychology cluster and the last one to the lifestyle cluster. The motivation KPI was by far the most important variable in the network, with a weight of 20%, followed by stress management, with a weight of 8.6%. Both belonged to the psychology cluster, and the reason for their importance might be their connection with the high-level strategic objectives defined in the present study. The definition and accomplishment of a dual career was also an important KPI and positively affected the motivation level of the judoka (Table 4).

The B class contained KPIs from the clusters of lifestyle, psychology, and specific training and none from the physical training cluster. The highest ranked KPI in the B class was 8/8/8 (8-h training, 8-h sleep, and 8-h rest per day), followed closely by focus and concentration level. This was aligned with the nutrition KPI, which was directly linked with the weight of the athlete and the severity of the discipline required for rapid weight loss in preparation for competitions. With regard to specific training, the Kumi-Kata KPI was the most important in the B class, followed by scouting and basic technique.

Finally, the C class included all of the KPIs with less global importance in the network: one in the psychology cluster, five in the specific training cluster, and all 10 in the physical training cluster.

From a global perspective (Table 6), three strategic objectives were in class A: qualification for the Olympic Games (10.3% of total importance), winning a Grand Prix or Grand Slam medal (8.9% of total importance), and an improvement in international ranking (with 4.4% of total importance). The final strategic objective (to become or remain a national number one) was a B-class objective (2.7% of total importance). Therefore, qualifying for the Olympic Games was by far the most important strategic objective of the judokas in the study, followed by winning either a Grand Slam or Grand Prix.

**Table 6:** Normalized-by-cluster matrix.

**Cluster**	**KPI**	**Normalized by cluster**
Lifestyle	8/8/8	0.35404
	Dual career (Dual)	0.41893
	Nutrition (Nut)	0.22702
Physical training	Aerobic resistance (AR)	0.09993
	Anaerobic resistance (ANR)	0.09251
	Cardiac frequency—basal (CF)	0.06895
	Cardiac recovery (CR)	0.05857
	Coordination (Coor)	0.07517
	Flexibility (Flex)	0.01638
	Maximum strength (MaxS)	0.17542
	Power (Pw)	0.17039
	Relative strength (RelS)	0.15677
	Speed (Sp)	0.0859
Psychology	Activation level (Act)	0.04429
	Focus and concentration level (Foc)	0.0967
	Motivation (Mot)	0.52381
	Stress (Stress)	0.22515
	Team cohesion (Cohe)	0.11004
Specific training	Age	0.00244
	Basic technique (BT)	0.11431
	Basic tactic (BTac)	0.13968
	Kumi-Kata (KK)	0.22303
	Scouting (SC)	0.17927
	Specific technique (ST)	0.11245
	Specific tactic (Stac)	0.11619
	Weight (Wei)	0.11262

To calculate the relative importance of each element of a cluster, the normalized-by-cluster matrix was obtained (Table 6).

In the psychology cluster, motivation was the most important KPI, with more than 50% relative importance, followed by stress, team cohesion, focus and concentration level, and activation level. The specific training cluster contained three leading KPIs—Kumi-Kata, scouting, and basic technique—while the other KPIs had equivalent importance, with the exception of age, which had nearly zero importance. In the physical training cluster, the most important KPIs were maximum strength, power, and relative strength, each of which had more than 10% relative importance. The other KPIs in the cluster amounted to <10% importance, with flexibility the least important. Finally, within the lifestyle cluster, dual career accounted for more than 40% relative importance, followed by 8/8/8 and nutrition.

## Discussion

We found that male half-lightweight judokas (under 66-kg category) aiming to participate in the Olympic Games, to win a medal in either a Grand Slam or Grand Prix, and to improve their international ranking had great motivation skills, stress management capacity, and an ability to strengthen their team's cohesion. These results were consistent with the superiority of personality traits and psychological skills of Olympic-level athletes reported in a systematic review of articles (published from 1984 to 2017) that dealt with the talent-related characteristics of outstanding athletes (Issurin, 2017). Some authors stated that coaches cited psychological training as a determining factor in judo competition success (Lane, 2007; Santos et al., 2015; Zurita-Ortega et al., 2017).

Motivation was by far the most important KPI of success in the present study. This is not surprising, since the link between motivation and sport performance in judo competition has been reported previously (Gillet et al., 2010). In particular, Gillet et al. (2010) indicated that judokas who displayed self-determined situational motivation toward competition performed better during the subsequent event. Moreover, such motivation was related with contextual self-determined motivation, and this in turn was significantly and positively related with autonomy-supportive coaching. This kind of coaching style is therefore warranted if high-level strategic objectives are to be achieved. It has also been found that mindfulness, rather than passion, is the main factor enhancing intrinsic motivation in athletes (Amemiya and Sakairi, 2019). Exercises to maintain and improve mindfulness should, therefore, be added to regular training tasks to ensure a positive effect on motivation.

Stress management was one of the class A KPIs. Psychological skills encompass a number of practical tools that enable athletes to manage competitive stress and self-regulation. These are fundamental in reaching reach peak performance, both preparation- and participation-wise (Issurin, 2017). Judokas would benefit for being in an environment that challenges their comfort zone, where psychological assistance in dealing with stress is offered and where there are opportunities to train with high-level competitors. Therefore, in combat sports and judo, the organization of international training camps where there are chances to fight with different fight styles with outstanding judokas is habitual. Additionally, focus and concentration level has been ranked highly as a determinant KPI for high-level success. Thus, coaches should design situational tasks such as simulated competitions to challenge the attention of judokas. This would help them to work on their focus and concentration. Furthermore, coaches could also assess the attention capacity of judokas and their progress using some of the specific tools and methods developed for this precise purpose (Mihailescu and Sava, 2013). In addition, the JEG considered that the dual career KPI, a component of the lifestyle cluster, could provide both a backup and a way of keeping the mind busy. This can complement the judo training. The dual career KPI most influenced motivation (9.8%), and thus, it is important to define a coherent plan that avoids conflicts of identity but does not interfere with the judoka's results (Kavoura and Ryba, 2020).

The specific training cluster was ranked in both B and C classes, which indicates that it discriminated less between high-level and outstanding athletes than psychological factors. Judo is a complex sport in coordinative terms; athletes spend many years mastering techniques to throw their opponents, control them in groundwork, and/or dominate them sufficiently to force the referee to impose a sanction on them. Therefore, when a certain level of performance is reached, judokas are assumed to be of high technical-tactical quality. This may have been the reason why specific training was not regarded as particularly decisive in achieving the more ambitious strategic objectives. The emphasis on stress management (as indicated by the JEG) enables outstanding athletes to produce greater technical-tactical performances in stressful competitive situations. Negative factors such as fear, anxiety, and insufficient self-regulation may have strong detrimental effects on preparations for the big event and/or on the day itself. Within the specific training cluster, the Kumi-Kata KPI was ranked highest by the JEG. Judokas invest approximately half of the total combat time, pauses not included, on Kumi-Kata (Marcon et al., 2010). It is seen as the *condition sine qua non*, because throwing techniques are not possible without proficiency in this area; judokas employ it to dominate the combat and to encourage the referees to sanction the opponent. This is why Barreto et al. (2019) proposed that coaches should introduce training tasks to improve the approaching moment in Kumi-Kata.

Finally, the JEG considered power and strength the most important KPIs within the physical cluster. This was in keeping with a great deal of other research on performance in judo (Franchini et al., 2014, 2019). Physical capacities were valued as essential in achieving high-level strategic objectives, but the JEG did not indicate that KPIs were discriminant indicators between the most successful judokas and those who do not reach the highest level in judo competitions. These results contrast with a recent investigation by Kons et al. (2020), where neuromuscular and judo-specific test differentiated the judo ranking position. However, the level of the strategic objectives studied by Kons et al. (2020) was lower (i.e., regional ranking) than the strategic objectives analyzed by the JEG in the present instance. The possibility that specific judo tests can discriminate between performance levels in young judokas—and could therefore be used as a tool to detect sport talent—has attracted some interest (Lidor et al., 2005). Lidor et al. (2005) monitored the fitness of 10 young male judokas over 2 years, but the outcomes did not reveal any predictive potential of the athletic ranking of the participants after a follow-up program was completed. As the authors indicated, this may have been due to the relatively low sensitivity of the general and specific test batteries used to detect differences between the highly specific judo skills.

The main strengths of the research are as follows: (i) the novel data analysis with the application of the ANP; (ii) the identification and prioritization of KPIs and strategic objectives for improving judokas' performance; (iii) the international and intercultural elite judo expert group formed; and (iv) the application of the methodology to an international high-performance judo center. On the other hand, the main limitations are as follows: (i) the results from applying the methodology are limited to the male under 66-kg judo category and (ii) then, there are no other studies available yet for comparing the obtained results with either female counterparts with similar weight (under 52 kg) or heavyweight judo athletes.

Our results demonstrate the importance of psychological KPIs in reaching the defined strategic objectives for high-level male half-lightweight judo athletes. Future studies should investigate whether these KPIs apply to female counterparts of similar weight or heavyweight judo athletes.

## Conclusions

The present study has outlined an easily replicable methodology by which to identify and prioritize the main KPIs affecting performance in elite-level judo. Then, regarding the two main objectives of the research, (1) to identify and to prioritize the main KPIs that can help to build on the high performance of professional judokas and (2) to establish the link between judo KPIs and the achievement of strategic objectives, the results of applying this methodology at the High-Performance Judo Centre of Valencia showed that, for the male under 66-kg category, the most important KPIs (out of the 26 identified) in reaching certain strategic objectives (i.e., participating in the Olympic Games or winning a medal in either a Grand Prix or a Grand Slam) were related to the psychological cluster. Motivation was by far the most important KPI, followed by stress and team cohesion. Regarding the third objective of the paper, to make recommendations to improve performance based on the findings, it is recommended that judokas should receive professional psychological support during daily training sessions, and coaching staff should foster international team gathering and sports internships. Other important KPIs were Kumi-Kata, dual career, focus and concentration level, scouting, nutrition, and basic technique. Finally, power and strength were the most important physical KPIs; however, while they were considered to be essential for high-level competitors, they did not make the difference between outstanding and high-level judokas. The JEG (which comprised five elite judokas, national and international champions, and three trainers, including a former European champion and a world championship silver medalist) added high value to the study.

## Data Availability Statement

The original contributions presented in the study are included in the article/Supplementary Material, further inquiries can be directed to the corresponding author/s.

## Ethics Statement

The studies involving human participants were reviewed and approved by Research Ethics Committee of the Universitat Politècnica de Valéncia. The patients/participants provided their written informed consent to participate in this study.

## Author Contributions

SU, RR-R, and J-JA-S made the definition of the methodology and conducted the analysis of results. SU, RR-R, J-JA-S, EC, and MU conducted the application of the methodology and wrote the manuscript. SM, RR-R, J-JA-S, and EC made the discussion. All authors contributed to the article and approved the submitted version.

## Conflict of Interest

The authors declare that the research was conducted in the absence of any commercial or financial relationships that could be construed as a potential conflict of interest.
