# Supplementary material for: Improving on Half-Lightweight Male Judokas' High Performance by the Application of the Analytic Network Process
Source: Front Psychol. 2021 Apr 7;12:621454. doi: 10.3389/fpsyg.2021.621454 (PMC8058178; doi:10.3389/fpsyg.2021.621454)
Supplement: Supplementary file 1 [file Data_Sheet_1.PDF]

As mentioned in the paper, the first step of the Analytic Network Process (ANP; Saaty, 1996) is to define the **Dependence matrix**, which indicates whether there are meaningful relationships between each pair of variables of the study or not. These relationships are indicated with a value of 1 in the correspondent intersections of each pair of variables in the matrix (Saaty, 1996). Additionally, the variables of the study are grouped in different clusters according to their common characteristics; e.g., the physical training KPI constituted the "Physical training cluster." Then, the intensity of the relationships identified in the Dependence matrix is quantified using a pair-wise questionnaire using the Saaty's Fundamental Scale (presented in the paper). From the results of these comparisons, a pairwise comparison matrix A is determined (Figure 1), whose eigenvector is worked out, which needs to be normalized in order to make sure that the sum of all its values is equal to 1 as well as to unify the scale (Saaty, 1996).

$$A = \begin{bmatrix} 1 & a_{12} & \cdots & a_{1n} \\ a_{21} & 1 & \cdots & a_{2n} \\ \vdots & & \ddots & \vdots \\ a_{n1} & a_{n2} & \cdots & 1 \end{bmatrix}, \text{where } a_{ji} = 1/a_{ij} \quad i,j = 1,\ldots,n$$

Figure 1: Generic pairwise comparison matrix (Saaty and Vargas, 2013)

Additionally, it is necessary to take into account that the intensity of the relationships between each pair of variables defined must be consistent and not contradictory ones, fact that is more feasible the bigger is the dependence matrix and the number of relationships between variables established. In this sense, it is used the called Consistency Ratio (CR), which is used to check and identify inconsistencies in the judgement of decision-makers when quantifying the relationships between variables. The CR is calculated as *CR=CI/RI*, where $CI = \frac{\lambda max - n}{n-1}$, and λmax is the maximum eigenvector of the pair-wise comparisons and n is an experimental value (Saaty, 1980). In this sense, Saaty (1994) set up the next treshold values: 0.08 for n=4 and 0.1 for $n \geq 5$.

The next step is, from the matriz A and once all the CR of each pair-wise comparison have been checked and, if necessary, corrected, to work out the **Unweighted matrix**. The Unweighted matrix shows the variables grouped in clusters and represents which element is more influencial, and to what extent, among the elements of a cluster

(Saaty, 1996). The Figure 2 presents a generic Unweighted matrix, where each column *Wij* is an eigenvalue of the influence of the elements in the i_{th} component over an element in the j_{th} component, and *n* the number of elements of the network. Then, some of the entries of the Unweighted matrix will be of null value, correspondent to each pair of variables with no influence between them. Additionally, it is necessary to consider that the priorities used in the calculus of the Unweighted matrix are local ones or normalized vectors of relative priorities between elements.

$$W_{ij} = \begin{bmatrix} W_{i1}^{(j1)} & W_{i1}^{(j2)} & \cdots & W_{i1}^{(jn_j)} \\ W_{i2}^{(j1)} & W_{i2}^{(j2)} & \cdots & W_{i2}^{(jn_j)} \\ \vdots & \vdots & \cdots & \vdots \\ W_{in_i}^{(j1)} & W_{in_i}^{(j2)} & & W_{in_i}^{(jn_j)} \end{bmatrix}$$

Figure 2: Generic Unweighted matrix (Saaty and Vargas, 2013)

Fom the Unweighted matrix is possible to obtain the **Weighted matrix**, which is a stochastic matrix that uses vectors of priorities between clusters to calculate the global importance of the variables respect to the rest of both variables and clusters of the network. The Figure 3 presents a generic Weighted matrix, which represents the result of multiplying the priority vectors between clusters by the correspondent blocks of the Unweighted matrix, obtaining then the coefficients of the Weighted matrix.

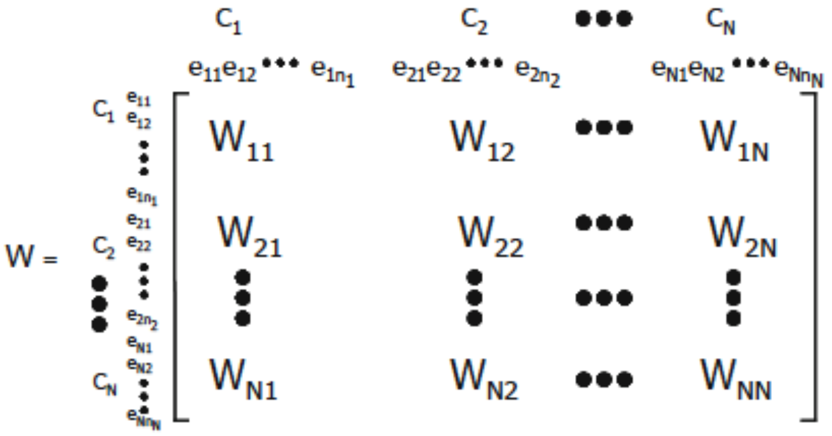

Figure 3: Generic Weighted matrix (Saaty and Vargas, 2013)

From the Weighted matrix it is possible to obtain the quantification of the cause-effect relationships between each pair of variables. Then, since the sum of the values of the

columns of the Weighted matrix is equal to 1, this means that a concrete value between a pair of variables directly offers, in percentage, the value of the cause (variable in the row) and effect (variable in the column) link between the two variables.
The next step is to work out the **Limit matrix**. In order to do it, the Weighted matrix is raised to as many powers as necessary until it stabilizes and finally converges, which takes place when all the columns have the same values. Then, the sum of all the values of each column will be equal to 1, and the weight of each variable will represent its global relative weight within the network; in other words, its global importance.

More information about the Analytic Network Process may be found at the references cited previously:

- Saaty, T.L. (1980). *The Analytic Hierarchy Process.* RWS McGraw-Hill.
- Saaty, T.L. (1994). *Fundamentals of Decision Making and Priority Theory With the Analytic Hierarchy Process*. first ed. RWS Publications, Pittsburgh.
- Saaty, T.L. (1996). *The Analytic Network Process: Decision Making with Dependence and Feedback*. RWS Publications, Pittsburgh, PA.
- Saaty, T.L., Vargas, L.G. (2013). *Decision making with the Analytic Network Process. Economic, Political, Social and Technological Applications with Benefits, Opportunities, Costs and Risks*. 2nd Edition. Springer Science+Business Media, New York, NY.
